# Transcriptomics, metabolomics and histology indicate that high-carbohydrate diet negatively affects the liver health of blunt snout bream (*Megalobrama amblycephala*)

**DOI:** 10.1186/s12864-017-4246-9

**Published:** 2017-11-09

**Authors:** Wassana Prisingkorn, Panita Prathomya, Ivan Jakovlić, Han Liu, Yu-Hua Zhao, Wei-Min Wang

**Affiliations:** 1 College of Fisheries Huazhong Agricultural University, Key Lab of Freshwater Animal Breeding, Ministry of Agriculture, Key Lab of Agricultural Animal Genetics, Breeding and Reproduction of Ministry of Education, Freshwater Aquaculture Collaborative Innovation Center of Hubei Province, Wuhan, 430070 People’s Republic of China; 2 Bio-Transduction Lab, Wuhan Institute of Biotechnology, Wuhan, 430075 People’s Republic of China

**Keywords:** Fish, Aquaculture, Hepatosomatic index, NAFLD, Neurodegenerative diseases

## Abstract

**Background:**

Global trend of the introduction of high levels of relatively cheap carbohydrates to reduce the amount of costly protein in the aquatic animal feed production has affected the aquaculture of an economically important cyprinid fish, blunt snout bream (*Megalobrama amblycephala*). This dietary shift has resulted in increased prevalence of metabolic disorders, often causing economic losses. High dietary intake of carbohydrates, associated with obesity, is one of the major causes of non-alcoholic fatty liver disease (NAFLD) in humans.

**Results:**

We have conducted an eight-week feeding trial to better understand how a high-carbohydrate diet (HCBD) affects the liver health in this fish. Hepatosomatic index and lipid content were significantly (*P* < 0.05) higher in the HCBD group. Histology results also suggested pathological changes in the livers of HCBD group, with excessive lipid accumulation and indication of liver damage. Metabolomics and serum biochemistry analyses showed that a number of metabolites indicative of liver damage were increased in the HCBD group. This group also exhibited low levels of betaine, which is a metabolite crucial for maintaining the healthy liver functions. Transcriptomic and qPCR analyses indicated that HCBD had a strong impact on the expression of a large number of genes associated with the NAFLD and insulin signalling pathways, which may lead to the development of insulin resistance in hepatocytes, pathological liver changes, and eventually the NAFLD.

**Conclusions:**

Transcriptomics, metabolomics and histology results all indicate early symptoms of liver damage. However whether these would actually lead to the development of NAFLD after a longer period of time, remains inconclusive. Additionally, a very high number of upregulated genes in the HCBD group associated with several neurodegenerative diseases is a strong indication of neurodegenerative changes caused by the high-carbohydrate diet in blunt snout bream. This suggests that fish might present a good model to study neurodegenerative changes associated with high-carbohydrate diet in humans.

**Electronic supplementary material:**

The online version of this article (10.1186/s12864-017-4246-9) contains supplementary material, which is available to authorized users.

## Background

Dietary carbohydrates are the main source of energy for herbivorous animals. Glucose and other monosaccharides obtained from the digestion of dietary carbohydrates are metabolized in the liver, the major site of carbohydrate metabolism and triglyceride synthesis, to provide precursors (such as acetyl-CoA) for the synthesis of ATP and fatty acids. Carbohydrates are usually the cheapest dietary source of energy, and their appropriate incorporation reduces the catabolism of more costly dietary proteins and lipids for energy. Hence the inclusion of high levels of carbohydrates is favored by the aquaculture industry, as it usually leads to a reduction in the cost of formulated diets [1–3]. However, the ability of fish to use dietary carbohydrates varies widely among and within species. Although the inclusion of high percentage of dietary carbohydrates appears to induce positive effects on growth and digestibility in several fish species [4–6], in comparison to mammals, fishes are generally considered to be poor utilizers of carbohydrates, with high dietary protein requirements and low tolerance to carbohydrates [7–10]. In herbivores (partial or predominant), though, if the appropriate level of carbohydrates is not included in the diet, there may be negative effects on the nutrient utilization, growth, metabolism, and health of the fish [11, 12]. In comparison to mammals, the source and the type of dietary carbohydrate have much higher impacts on the utilization of carbohydrates in fish. For example, dietary glucose is known to result in reduced growth performance and feed utilization in comparison to dietary starch in several fish species [2, 13, 14]. In mammals, over-ingestion of simple carbohydrates, such as sucrose and fructose, is a major cause of nonalcoholic fatty liver disease (NAFLD) [15]. Although high dietary intake of carbohydrates has been associated with increased risks of obesity in fish, and zebrafish and medaka were used as models to investigate whether fish livers are affected by excessive dietary carbohydrates, including changes in liver size, cellular architecture, gene expression patterns, and lipid accumulation, the correlation between high carbohydrate diet and NAFLD in fish remains inconclusive [16–18].

Blunt snout bream (*Megalobrama amblycephala*), a predominantly herbivorous cyprinid fish native to China, has rapidly joined the ranks of the handful of economically most important fishes in Chinese freshwater aquaculture during the last two decades [19, 20]. As it was domesticated barely more than half a century ago from a very small founding population [21], with the rapid development of the aquaculture of this species, founder effect (reduction in genetic diversity) has contributed to the increasing appearance of growth depression, early maturation [22] and low disease resistance in cultured populations [23]. The global trend of introduction of high levels of relatively cheap carbohydrates and fat in order to reduce the amount of costly protein in aquatic animal feed production has affected the aquaculture of this species as well [3, 24–26]. This dietary shift has resulted in increased prevalence of metabolic disorders, such as high deposition of body lipids and weakened immune system, in many farmed fish species, including the blunt snout bream. These disorders often result in high mortality and low market quality of the farmed fish, causing economic losses to the industry [11, 26, 27].

Few studies that attempted to examine the impacts of HCBD on blunt snout bream have produced inconclusive results: Li et al. [26] found that this fish utilizes dietary lipids better than carbohydrates; but Li et al. [28] found that high dietary lipids cause metabolic stress, whereas HCBD did not produce any adverse results; Zhou et al. [27], however, found that HCBD causes the swelling of hepatocytes. The latter is in agreement with the results of our previous study, where we found that high-carbohydrate diets caused signs of liver damage [29].

Therefore, to contribute to a better understanding of the NAFLD in the blunt snout bream (essential for the aquaculture of this species) and fish in general, we have investigated how a high carbohydrate diet impacts the gene expression in blunt snout bream by sequencing the entire transcriptome. To provide additional focus on the NAFLD indices, we have also studied the liver histology, levels of some metabolites and expression of specific genes associated with the NAFLD.

## Methods

### Diet preparation and proximate analysis

Two diets (Table 1) were formulated to contain approximately 30% proteins and 8% lipids. The control diet was formulated according to the current blunt snout bream aquaculture industry standards (25–30% carbohydrates) [30, 31] to contain 26.6% carbohydrates. As Zhou et al. have proposed that diets containing 31–47% carbohydrates cause a stress response in blunt snout bream [27, 31], the HCBD was formulated to contain over 34% of carbohydrates (Table 1). As a result, the total energy was higher in the HCBD diet (23.5 vs. 19.1 MJ/kg). The ingredients (Table 1) were powdered, mixed, pelleted, air-dried, then oven-dried (105 °C) overnight, sealed in airtight bags, and stored at −20 °C until use. The crude protein, crude lipid, moisture, and ash in the feeds were determined using standard methods [32]: moisture by oven-drying at 105 °C, ash using a muffle furnace at 550 °C, crude protein (N × 6.25) using the Kjeldahl method after acid digestion with the Kjeltec system (Kjeltec 2300 Analyzer, Foss Tecator, Hoganas, Sweden), crude lipid using the ether extraction method with the Soxtec System HT (Soxtec System HT6, Tecator). Energy contents of the diets were measured with a bomb calorimeter Parr 6200 equipped with a Parr 1108 Oxygen Bomb and a Parr 6510 water handling system (Parr Instrument Company, Moline, IL, USA).

**Table 1:** Formulation and proximate composition of the two diets

	Control	HCBD
Ingredient		
Soybean meal	35.50	34.00
Rapeseed meal	19.00	19.00
Wheat bran	1.20	1.20
Fishmeal	8.00	8.00
Wheat flour	18.00	26.30
Soybean oil	3.80	5.20
Lysine	0.30	0.30
Ca(H_2_PO_4_)	2.00	2.00
Premix^a^	1.00	1.00
Choline chloride	1.00	1.00
Carboxymethyl cellulose	2.00	2.00
Zeolite	8.20	0.00
Proximate composition (%)		
Protein	30.84	30.28
Lipid	7.99	8.26
Carbohydrate	26.63	34.13
Ash^b^	16.61	8.78
Moisture	13.42	12.61

^a^Multivitamin and mineral premix for the herbivorous fish (Wuhan Haid Group Co., Ltd): 0.5% multivitamins and 0.5% minerals. ^b^Because we used a high level of zeolite minerals (8.20%) in the control diet, the total ash is also much higher

### Animal rearing, experimental procedures and sample collection

Healthy one-year-old blunt snout breams (*n* = 270; average weight = 48.13 ± 0.02 g) were obtained from the experimental base of the Huazhong Agricultural University, Wuhan, China, distributed into six aerated fiberglass tanks (45 individuals per half-filled 1 m^3^ tank) and fed the control diet during the acclimation period (14 days). At the onset of the experiment, three tanks were randomly assigned to two different experimental diet groups: control and HCBD (each *n* = 135). Three tanks in each group (3 × 45 = 135) ensured three replicates per diet. The period of the rearing trial was the standard [33] 8 weeks, during which the water temperature was 25 ± 0.5 °C and the photoperiod was natural. Between 25 and 50% of the water was replaced in each tank daily. The fish were fed by hand twice daily. At the end of the experiment, after a 12-h fast, all of the surviving fish (control = 128, HCBD = 132) were tranquilized in 20–30 mg/L MS-222 buffered to pH 7.0–7.5, and weighed pooled together. Five individuals from each tank were then randomly selected and euthanized in buffered MMS at 300 mg/L concentration. After weighing, blood was collected from the caudal vein using 1 mL syringes and plasma samples prepared according to the standard protocol for clinical chemistry measurements, using sodium heparin as anticoagulant. All blood samples were centrifuged immediately after collection and supernatant (plasma) stored at −80 °C. After dissection, entire livers were collected and weighed in order to calculate the hepatosomatic index (HSI). A small portion of each liver was immediately fixed by submerging into 1 mL of paraformaldehyde in a 2 mL Eppendorf tube for histopathological examination by H&E and Oil red O staining [34], whereas samples for transcriptomic and metabolomic analyses were placed in 2 mL plastic tubes, immediately flash-frozen in liquid nitrogen for 6 h, and stored at −80 °C. Liver lipid content was calculated from the Oil red O staining results using Image-pro plus 6.0 (Media Cybernetics, Inc., Rockville, MD, USA). For details regarding determining the growth performance, please see our previous publication [29]. Data were analyzed statistically with SPSS 20 (IBM, Chicago, IL, USA), where statistical differences between the two groups were determined using the unpaired two-tailed analysis (*t* test). Thresholds for statistical significance were set at *P* < 0.05 (significant) and *P* < 0.01 (highly significant). Results were expressed as means ± SD.

### Metabolomic analysis

As we have studied the metabolome and serum biochemistry parameters in detail in our previous study [29], here we have focused exclusively on the parameters indicative of liver damage [28]. Among the 30 collected serum samples, ten samples were chosen randomly from each group for clinical biochemistry and metabolomic analyses. Hence, ^1^H NMR metabolomic analysis was conducted only for betaine in liver samples (not published before), whereas for relevant metabolites in serum samples (α-glucose, β-glucose, succinate and tyrosine), as well as the serum biochemistry parameters (alanine transaminase - ALT, aspartate transaminase - AST, triglycerides - TG, high-density lipoprotein - HDL and low-density lipoprotein - LDL) we referred to the appropriate data (HCBD group) from the previous study.

#### Clinical biochemistry

Serum biochemistry parameters were measured with commercial kits produced by Jiancheng Bioengineering Institute (Nanjing, China) with a Tecan analyzer (Tecan Ltd) as described before [29].

#### Nuclear magnetic resonance (^1^H–NMR) spectroscopy

Conducted as described before [29]. Briefly: liver tissues (about 50 mg) were homogenized in cold methanol and water (*v*/v = 2:1) using a Qiagen TissueLyser (Retsch GmBH, Germany). All plasma NMR spectra were acquired at 298 K on a Bruker Avance III 600 MHz NMR spectrometer (600.13 MHz for ^1^H frequency) equipped with a cryogenic probe (Bruker Biospin, Germany). One-dimensional ^1^H NMR spectra were acquired with the Carr–Purcell–Meiboom–Gill pulse train [35]. The NMR spectroscopic analysis, NMR data processing, and multivariate data analysis were performed as previously described [36]. The cutoff value (|r| > 0.602) was based on the significance threshold value of *P* < 0.05, which was determined according to the test for significance of the Pearson’s product-moment correlation coefficient (*n* = 10, *P* < 0.05).

### Transcriptome analysis

Among the 30 collected liver samples, one sample from each tank (3 control +3 HCBD) was randomly selected for the transcriptome sequencing, and sent (in liquid nitrogen) to the Biomarker Biotechnology Corporation (Beijing, China). The total RNA was extracted, cDNA libraries constructed and sequenced, and transcriptome assembled and annotated, as described before [37], so only a brief overview is given here:

- *RNA extraction*: the total RNA was extracted using RNAiso Plus Reagent (Takara Bio Inc., Dalian, China) according to the manufacturer’s instructions. The quality of the total RNA from the individual tissue samples was evaluated using electrophoresis in 1% agarose gels. The RNA was quantified spectrophotometrically with NanoDrop 2000 spectrophotometer (Thermo Scientific, Delaware, USA) and Agilent Bioanalyzer 2100 (Agilent, Santa Clara, CA).
- *cDNA library preparation and Illumina sequencing:* high-quality RNA (5 μg, 100 ng/μL) was used for the preparation of RNA-seq libraries using the Illumina HiSeq™ 2500 platform according to the manufacturer’s manual [38] (Biomarker Technologies Co., Ltd., Beijing, China). All raw transcriptome data have been deposited in the NCBI Short Read Archive (SRA) with the accession numbers SRR5763110, SRR5763111, SRR5763112, SRR5763116, SRR5763117 and SRR5763107.
- De novo *assembly of sequencing reads:* raw reads of the transcriptome datasets (control and HCBD) were cleaned by filtering out adaptor-only reads (nt length of the recognized adaptor ≤13 and the remaining adaptor-excluded nt length ≤ 35) and low-quality reads: reads in which more than 80.01% of the bases had a Q value of ≤ 30 were filtered out with the Fastq_filter software (Biomarker Technologies Co., Ltd). The clean reads were then assembled with Trinity platform [39] using three implemented modules, Inchworm, Chrysalis, and Butterfly, with the following parameters: ‘K-mer = 25, and group pairs distance = 300. Redundant sequences were eliminated, and the longest transcripts were recognized as unigenes, which were grouped together for the final assembly and subsequent annotation.
- *Annotation:* Open reading frames (ORFs) of transcripts and unigene sequences were predicted by the TransDecoder package integrated into Trinity, with the minimum ORF length set at 100 bp. Unigene reads were queried in a number of databases: NCBI’s BLASTn and BLASTx (Ref-Seq nr); as well as UniProtKB/Swiss-Prot (manually annotated and reviewed) and UniProtKB/TrEMBL (automatically annotated and not reviewed) [40]. Each unigene sequence was allocated a gene name according to the BLAST hit with the highest score. Getorf program implemented in EMBOSS software package (version 1.20.0) [41] was employed to predict the open reading frames (ORFs) and to select the longest ORF for each unigene using default parameters.
- *Ontology analysis:* these unigenes were used to query a series of databases, using BLASTx with the E-value cutoff = 1e^−5^. Blast2GO program [42] was used to produce Gene Ontology (GO) annotations. COG [43] and KEGG [44] databases were also queried. KEGG Orthology (KO) assignment was obtained using the KAAS server (bi-directional best hit method) [45]. KEGG enrichment analysis was conducted using KOBAS software [46] to statistically test the enrichment of differentially expressed genes in KEGG pathways.
- *Gene expression analysis:* The numbers of reads produced by the RNA-Seq analyses were normalized to reads per kilobase of transcripts per million (RPKM) to compute the gene expression levels [47]. Detection of differentially expressed genes was performed by EBseq software [48] using pairwise comparison. Benjamini-Hochberg procedure [49] (α < 0.01) was used to control for the false discovery rate (FDR). Genes were defined as differentially expressed (DEG) when they exhibited the following parameters: FDR < 0.001 and |log2 ratio| > 1 (the RPKM value of a gene in one sample is at least two-fold higher than in another sample).

### qPCR analysis

This method was used to further investigate the expression of 13 genes associated with the NAFLD pathway (Additional file 1) (http://www.kegg.jp/kegg/pathway.html) that were significantly differentially expressed in response to HCBD in the transcriptomic results. The total RNA was extracted and cDNA prepared, from the same six liver samples that were used for the transcriptome sequencing, as described in the previous section. The cDNA libraries were serially diluted 10-fold and used as templates for qPCR with the primers listed in Additional file 2. Primers were designed using Primer Premier 5 software (Premier Biosoft, USA) [50] on the basis of the sequences of these 13 genes obtained from the transcriptome data, and synthesized by the Tsingke Biological Technology company (Co. Ltd., Wuhan). qPCR was performed with LightCycler®480 II (Roche Diagnostics GmbH, Germany) and SYBR® Premix Ex Taq™ (Takara Bio Inc.) according to the manufacturer’s instructions. In brief: the total qPCR mixture reaction volume of 10 mL contained 5 mL Light-Cycler480 SYBR Green I Master, 3.6 mL ddH2O, 0.4 mL of forward and reverse primers and 1 mL of cDNA template. PCR procedure was as follows: pre-incubation at 95 °C for 5 min, followed by 45 cycles of 10 s at 95 °C, 10 s at the temperature adjusted to the specific primer pair (Additional file 2), and 10 s at 72 °C. Melt curve analysis was performed at the end. Reactions were performed in triplicate for each sample. Expression levels of the target genes were normalized to the expression levels of two reference genes: *18S* rRNA and *Rpl13a* [51], and expressed as fold changes relative to the target gene expression in the control group using the 2^–ΔΔCt^ method [52]. The qPCR data were analyzed statistically using Microsoft Excel and SPSS. Student-T test, implemented in SPSS, was applied to test the statistical significance of the differences between the two groups, where significance thresholds were set at *P* < 0.05 (significant) and *P* < 0.01 (highly significant).

## Results

### Hepatosomatic index and liver histology

**Fig. 1:**
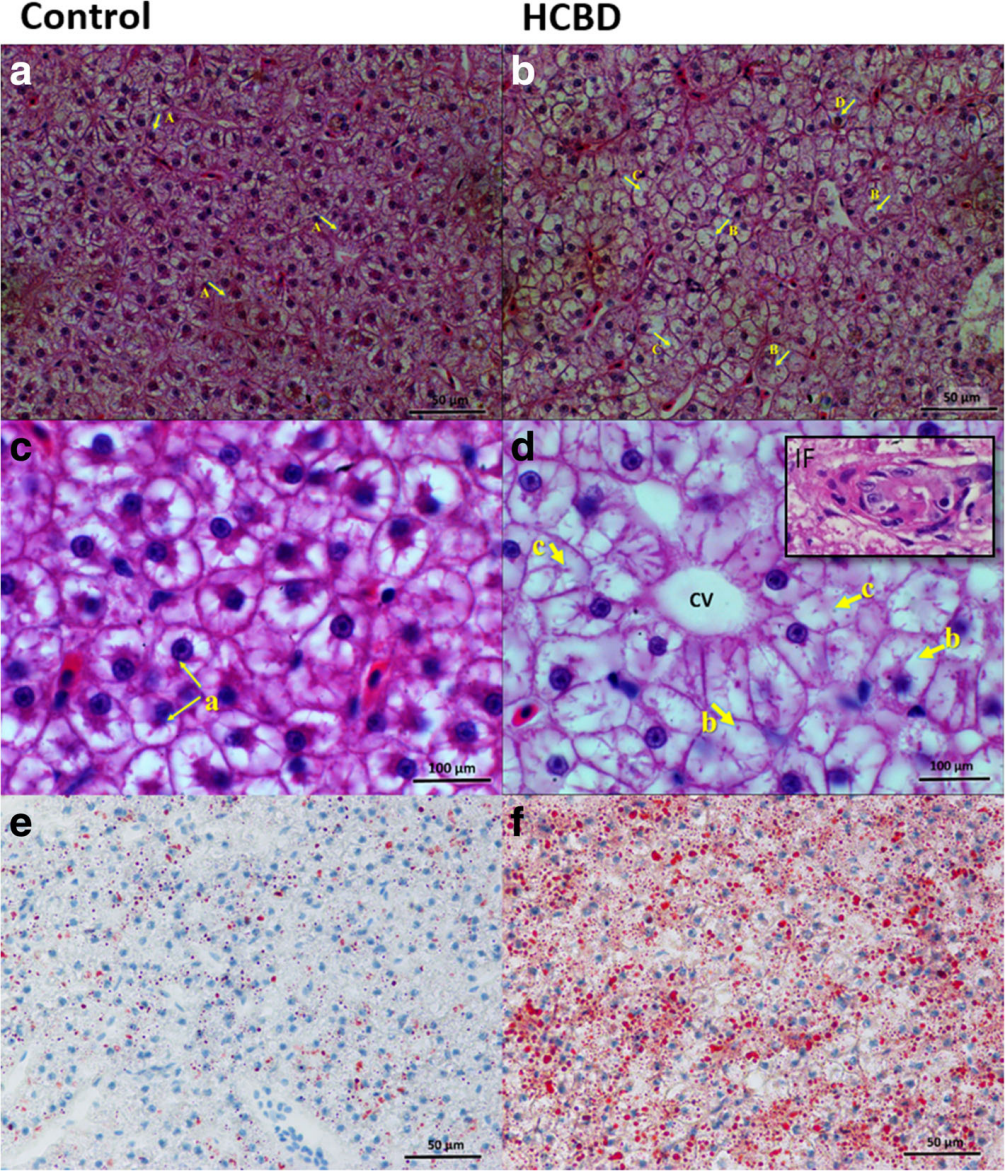
Photomicrographs of representative hematoxylin- and eosin-stained (**a**-**d**) and Oil red O-stained (E and F) histological liver sections of blunt snout bream fed control diet (**a**, **c** and **e**) and high-carbohydrate diet (**b**, **d** and **f**). Arrows indicate examples of a: normal hepatocytes with regular, round nuclei; b: swollen hepatocytes with large diffused lipid vacuoles, c: absent nucleus and d: abnormal nucleus. CV and IF labels indicate examples of central liver vein with abnormal endothelial cells and inflammatory infiltrate respectively. In the Oil red O-staining section, to evaluate lipid content in livers (**e** and **f**), lipids are stained red. Magnification ×400 (**a**, **b**, **e** and **f**) and ×1000 (**c** and **d**)

After the 60-day feeding period, survival rates of both groups were very high. Although the difference was not statistically significant (*P* = 0.530), survival rate of the HCBD group was higher: 97.78 ± 2.22 vs. 94.82 ± 7.14 (of the control group). On the contrary, HSI values were significantly (*P* = 0.020) higher in the HCBD group: 1.52 ± 0.13 vs. 1.40 ± 0.14. Liver lipid content and histology analyses have revealed the cause for the significant difference in the two HSI values: the control group had regular hepatocytes with large and spherical nuclei centrally located in a moderate cytoplasm and a small number of lipid droplets (Fig. 1a, c); in contrast, the HCBD group exhibited swollen hepatocytes with large diffused lipid vacuoles, abnormal endothelial cells in the central liver vein, inflammatory infiltrate and some hypertrophy of the hepatocytes (Fig. 1b, d). Moreover, significant fat accumulation in livers of the HCBD group was observed using the oil red O staining method (Fig. 1e, f): the lipid content in livers of the HCBD group was significantly (*P* = 0.006) higher than in the control group: 19.14 ± 2.30% vs. 3.95 ± 0.37% respectively.

### Serum biochemistry

**Fig. 2:**
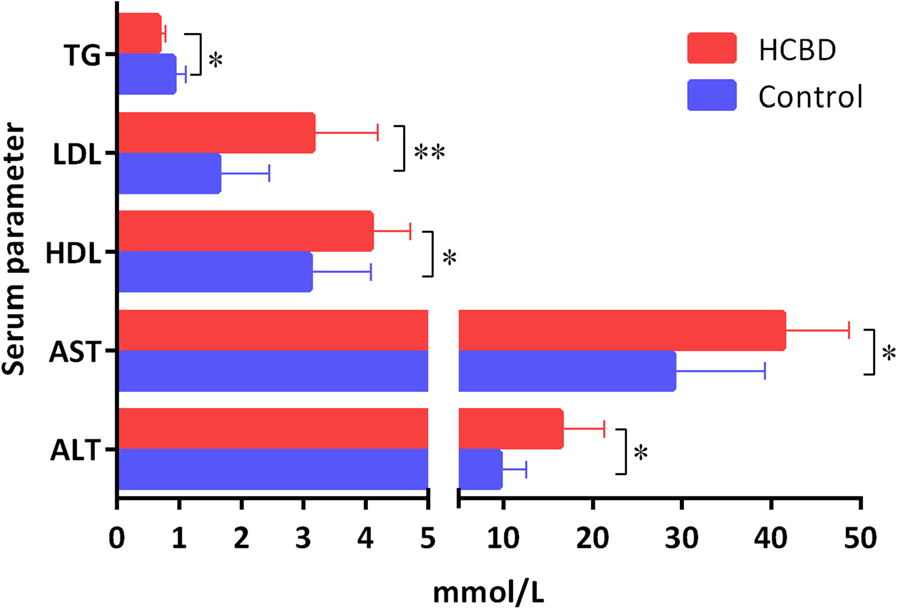
Serum biochemistry parameters associated with liver damage. * above bar indicates significant difference (*P* < 0.05) and ** indicates highly significant difference (*P* < 0.01). Results were analyzed by t-test

The HCBD has induced changes in all five studied serum biochemistry parameters: apart from TG, which was significantly reduced (*P <* 0.05), the remaining four parameters (AST, ALT, LDL, and HDL) were all significantly (LDL highly significantly *P <* 0.01) elevated in the HCBD group in comparison to the control group (Fig. 2).

### The metabolomics of serum and liver

**Fig. 3:**
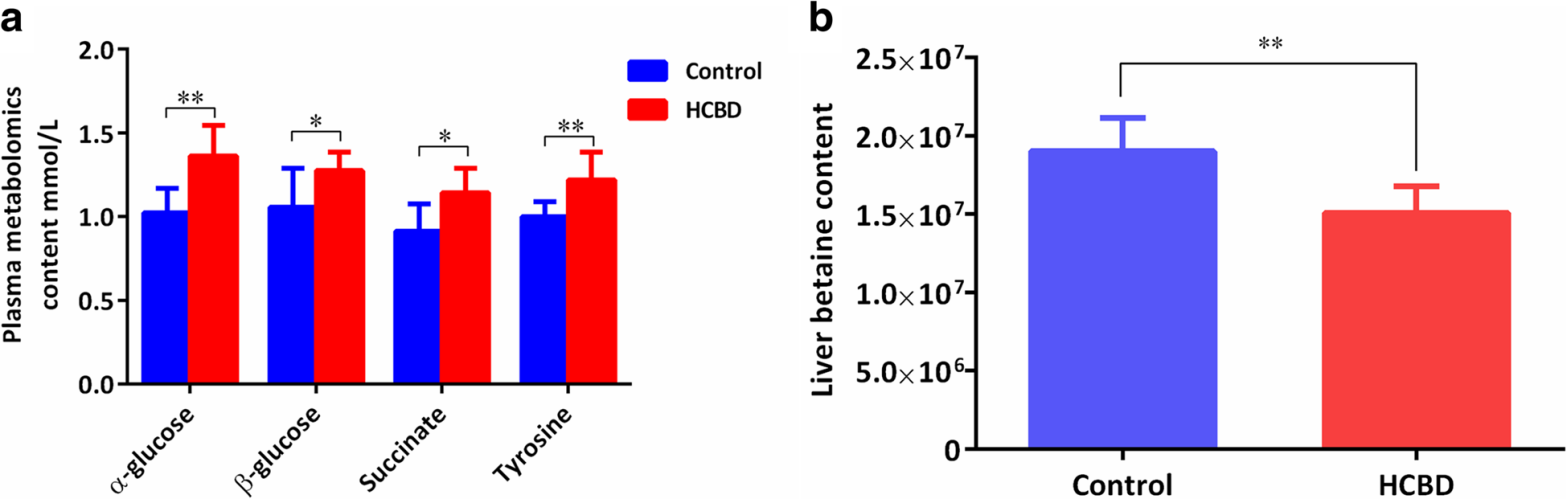
Metabolites in plasma and liver extracts significantly different between the control and HCBD diet groups. **a** Plasma metabolomics and **b** Liver metabolomics. Results were analyzed by t-test. * above bars indicates significant difference (*P* < 0.05) and ** indicates highly significant difference (*P* < 0.01)

The studied metabolites, indicative of liver damage, with significantly different abundance in the blood sera and livers of the control and HCBD groups, inferred from ^1^H–NMR metabolite profiles, are shown in Fig. 3. Metabolites belonging to two different metabolic pathways were (highly) significantly more abundant in the HCBD group. In glycolysis pathway, it was α-glucose (*P <* 0.01) and β-glucose (*P <* 0.05), and in tricarboxylic acid (TCA) cycle pathway it was succinate (*P <* 0.05) and tyrosine (*P <* 0.01) (Fig. 3a, Additional file 3). In liver, betaine was highly significantly reduced in the HCBD group (*P <* 0.01; Fig. 3b **,** Additional files 3, 4 and 5).

### Impacts of high-carbohydrate diet on the transcriptome

**Fig. 4:**
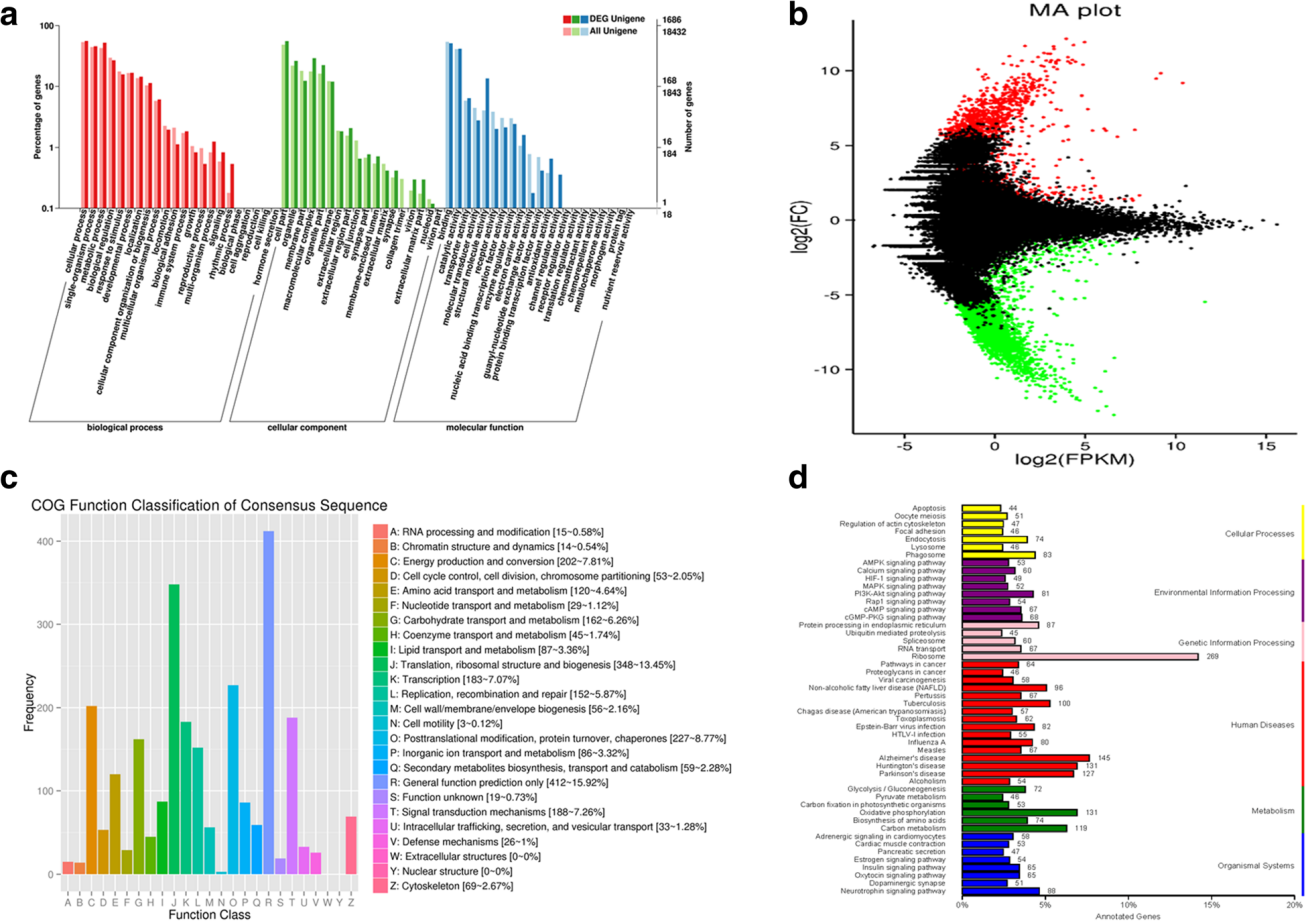
Transcriptome profiles of the HCBD and control groups. **a** GO annotation of all unigenes and DEG (differentially expressed) unigenes; **b** MA plot of differential gene expression levels in the two groups, where expression intensity is on the x-axis (log2 FPKM) and differences in the gene expression levels (fold change) are on the y-axis (log2 FC), each dot represents one gene, the log2 (FC) is plotted against the mean expression level for each gene, red dots represent genes whose abundance is significantly up-regulated, green dots - down-regulated, and black dots unchanged (or non-significantly changed) regulation; **c** COG annotation of and DEGs; **d** KEGG annotation of and DEGs

Non-normalized cDNA libraries were constructed from six tissue samples (3 control +3 HCBD) and Solexa/Illumina paired-end sequencing yielded 157,063 transcripts and 96,427 unigenes. After stringent quality assessment and data filtering, 22,415,280 high-quality reads from the control group and 19,837,027 from the HCBD group were selected for further analysis. GC contents of the control and HCBD samples were 47.76 and 47.80%, respectively. Using the GO annotation to assign all 96,427 unigenes to the three main categories, those assigned to the ‘molecular function’ (14,901, 36.56%) and ‘biological processes’ (14,583, 35.78%) were the most abundant, with ‘cellular components’ (11,271, 27.66%) lagging behind (Fig. 4a). Within the ‘biological processes’ category, the most frequent GO terms were ‘cellular process’, ‘metabolic process’, and ‘single-organism process’; among the ‘cellular components’, genes involved in ‘cell part’, ‘organelle’ and ‘macromolecular complex’ functions were the most abundant; whereas in the category of molecular function, the term ‘binding’ accounted for the highest proportion of annotations, followed by ‘catalytic activity’ and ‘molecular transducer activity’.

Among the 96,427 unigenes, 5556 significantly differentially expressed genes (DEGs) were identified by pairwise comparison of the HCBD with the control group; 1318 of them were upregulated and 4238 were downregulated in the HCBD group (Fig. 4b). Among these, 4440 were successfully annotated: 1935 using the COG database, 1686 using the GO database, 1895 using the KEGG database, 3973 using the Pfam database, 2294 using the Swiss-Prot database, and 3265 using the NR database.

Distribution of the 1686 genes successfully annotated using GO database in the three GO categories generally followed the pattern observed among all annotated unigenes (Fig. 4a), with some notable exceptions: in the ‘biological process’ category, genes associated with metabolic processes were more abundant (second, vs third most abundant), as were genes associated with multi-organism processes, signaling and rhythmic process, whereas biological adhesion and reproductive process categories exhibited reduced abundance. In the ‘cellular component’ category, the most notable was the complete absence of collagen trimer-associated DEG unigenes. In the ‘molecular function’ category, most notable was a strong increase in genes associated with structural molecule and channel regulator activities, whereas guanyl-nucleotide exchange factor activity-associated unigenes were notably reduced.

Among the 1935 DEGs successfully annotated using the COG database (Fig. 4c), the major group was ‘General function prediction only’ (412 unigenes; 15.92%), followed by ‘Translation, ribosomal structure, and biogenesis’ (348 unigenes; 13.45%), ‘Posttranslational modification, protein turnover, chaperones’ (227 unigenes; 8.77%), and ‘Energy production and conversion’ (202 unigenes, 7.81%). In ‘cell mobility’ category, only three unigenes were differentially expressed, and none in ‘extracellular structures’ and ‘nuclear structure’ categories.

To further clarify the biological pathways affected by the HCBD, the 1895 DEGs were mapped with the KEGG Pathway tools, and assigned to a total of 319 pathways, divided into six KEGG categories (Fig. 4d): the most strongly represented functional category was ‘Human Diseases’, containing 709 unigenes, the highest number of which (145) were associated with Alzheimer’s disease. Importantly, a large number (96) of DEGs in this category were also associated with NAFLD. Expectedly, a large number (592) of DEGs were associated with six different metabolic processes in ‘Metabolism’ category, with OXPHOS and Carbon metabolism at the top. By far the highest number (269) of DEGs was associated with Ribosome in the ‘Genetic Information Processing’ category (Fig. 4d, Additional file 6).

### qPCR

**Fig. 5:**
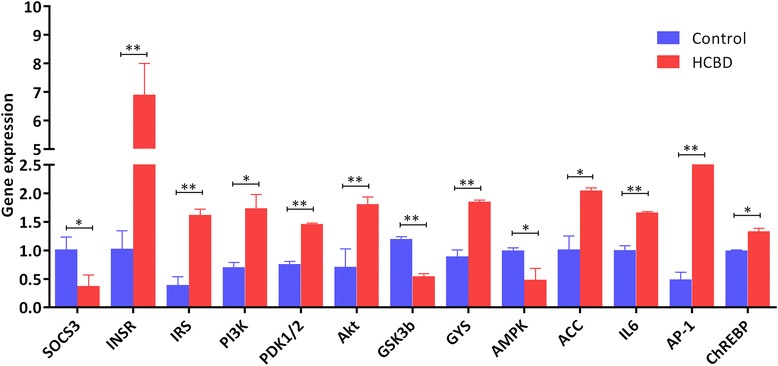
Expression of 13 DEGs from the transcriptome analysis associated with NAFLD, studied by qPCR. Data were normalized to *18 s* rRNA and *Rpl13a* as reference genes and presented as a fold change between the control and HCBD groups (mean ± SE). HCBD is the high-carbohydrate diet group. Results were analyzed by t-test. (*) above bar indicates significant (*P* < 0.05) and (**) highly significant differences (*P* < 0.01)

Among the 13 DEGs associated with the NAFLD pathway and Insulin signaling pathway (Additional files 1 and 7) (http://www.genome.jp/kegg/) studied by qPCR, ten were (highly) significantly upregulated in the HCBD group (in comparison to the control group), whereas three were (highly) significantly downregulated (Fig. 5).

## Discussion

### Hepatosomatic index and liver histology

The observed lipid vacuolization of hepatocytes and increased HSI indicate that the HCBD has resulted in de novo lipid synthesis [9, 53] and lipid accumulation in livers. Abnormal hepatocytes and lipid accumulation have been observed previously in association with high-carbohydrate diet in blunt snout bream [27], as well as in a number of other fish species, e.g., Nile tilapia (*Oreochromis niloticus*), gilthead sea bream [54], juvenile yellow catfish (*Pelteobagrus fulvidraco*) [55], juvenile *Labeo rohita* [56], and *L. rohita* fry [57]. High lipid accumulation was also the most likely cause for the high HSI value observed in the HCBD group. Increased HSI in association with high-carbohydrate diet was also observed in a number of other fish species, including golden pompano (*Trachinotus ovatus*) [58] and gilthead sea bream (*Sparus aurata*) [59, 60]. Therefore, high carbohydrate intake has caused fat accumulation and inflammatory infiltration in the liver, both of which are indicative of impaired liver health and precursors of the fatty liver disease [61–63].

### Serum biochemistry

High ALT and AST levels are indicators of liver damage and are associated with NAFLD [64, 65]. We hypothesize that an explanation for the much higher LDL levels observed here might be increased cholesterol synthesis in the liver in association with high dietary carbohydrates, resulting in an increase in the amount of LDL required to transport this cholesterol to other tissues. High levels of LDL are associated with obesity in mammals [66], and are generally a predictor of several chronic diseases [67]. LDL is also one of the causes of hepatic inflammation, and most importantly plays a critical role in the development of NAFLD [68]. Increased HDL levels in the HCBD group are somewhat confusing in this context, as these two metabolites are generally in negative correlation [69, 70]. The observed increase in both LDL and HDL levels may therefore indicate a general disorder in lipid metabolism caused by high de novo lipid synthesis. Also somewhat surprising was the lower level of triglycerides in the serum of the HCBD group, despite the high lipid accumulation in liver and marginally higher body weight [29]. However, TG levels in serum are driven by a large number of factors, and thus not particularly informative [71]. Liver dysfunction, attributable to liver damage associated with fatty liver, may be a part of the explanation for this phenomenon, insofar as the capacity of a damaged liver to pick up the extra TGs from the serum might be somewhat impaired. Additionally, low serum TGs have been associated with low betaine levels in mice [72], which was observed in the metabolomic analysis in our study (discussed in more detail in the following section). All these results are in agreement with previous observations that HCBD causes a metabolic stress response in blunt snout bream [31].

### The metabolomics of serum and liver

A part of the increase in HSI in the fish fed the HCBD may hypothetically be attributed to increased glycogen deposition in liver [1, 59, 73]. A previous study has also shown that blunt snout breams fed a high-carbohydrate diet had significantly higher hepatic glycogen content than those fed a control diet [27]. Our metabolomics results show that the high dietary carbohydrate intake induced significant increases in plasma α/β-glucose, succinate and tyrosine (Fig. 2), all of which can also be converted to acetyl-CoA in the TCA cycle pathway, which connects the glucose metabolism with the fatty acid metabolism. Therefore, although it is possible that the excessive carbohydrates available in the diet may have resulted in increased storage of α/β-glucose as glycogen, a large increase in the lipid content in the livers of the HCBD group suggests that it is much more likely that the majority of the superfluous carbohydrates were redirected to de novo lipid synthesis in the liver.

It is known that a decline in betaine is correlated with lower serum TGs in a diabetic mouse model [72]. Therefore, lower serum TG levels in the HCBD group are also consistent with the liver metabolomics results, where betaine was significantly reduced in the HCBD group. Betaine is the product of the irreversible oxidation of choline in liver and kidney. It can effectively prevent fructose-induced NAFLD and improve liver function by inhibiting inflammatory factors, reducing lipid peroxidation, reducing endoplasmic reticulum stress, and preventing apoptosis [74–76]. This indicates that a decline in liver betaine may be a consequence of the pathological hepatic changes associated with the high carbohydrate diet.

### Impacts of high-carbohydrate diet on gene expression

Thirteen DEGs were directly associated with the NAFLD pathway and insulin signaling pathway (Additional file 1), and their expression was further studied by qPCR (Fig. 5). The expression of the *suppressor of cytokine signaling 3* (*SOCS3*) gene, which plays an important role in the pathogenesis of obesity in animal models [77], was downregulated (*P <* 0.05). This gene correlates negatively with the *insulin receptor (INSR)* and the *insulin receptor substrate (IRS)* [78], both of which were accordingly upregulated (*P <* 0.01). *INSR* is a member of the receptor tyrosine kinase family, which binds insulin and other ligands that regulate glucose uptake and release, as well as the synthesis and storage of carbohydrates, lipids, and proteins [79]. Insulin can also regulate its own expression by binding to cell-surface INSR and stimulating phosphorylation-dependent signaling cascade [80]. IRS phosphorylates the P13 kinase, which was significantly upregulated in the HCBD group (Fig. 5), by binding to its regulatory subunit p85 [81] and thereby generates Phosphatidylinositol-(3,4,5)-triphosphate (PIP3), which in turn leads to the activation of several PIP3-dependent serine/threonine kinases, including PDPK1 and subsequently AKT/PKB [79, 82].

**Fig. 6:**
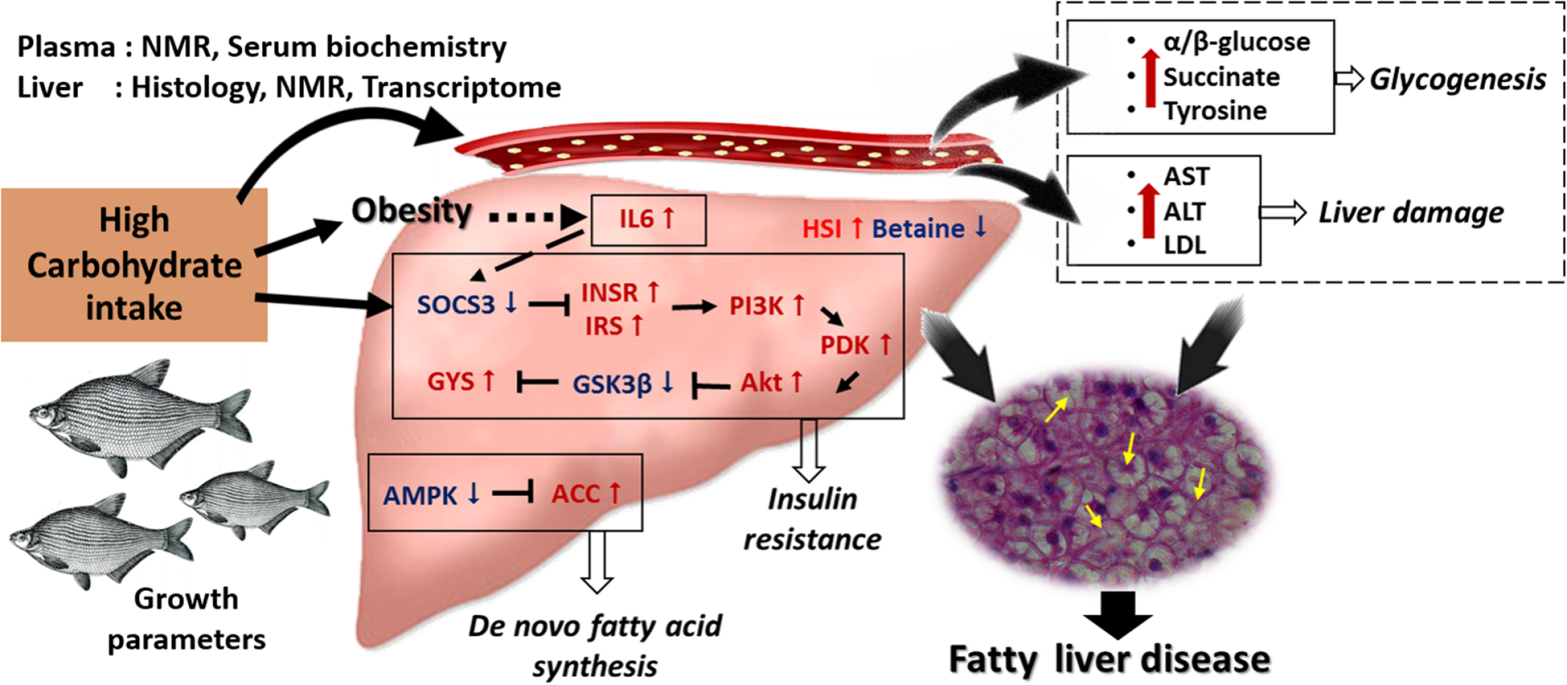
A hypothetical mechanism through which the observed changes in transcriptome, serum biochemistry, and serum and liver metabolomics caused by high-carbohydrate diet can lead to fatty liver disease

Upregulation in the expression of *INSR* and *IRS* genes has further led to upregulation of the downstream elements in this signaling pathway (Fig. 5, Additional file 1): *phosphoinositide-3-kinase (PI3K)*, *phosphoinositide-dependent protein kinase 1 (PDPK1)*, and *RAC-gamma serine/threonine-protein kinase (AKT)* in the HCBD group (although the transcriptomic data show downregulation of *PI3K*, see Table 2, qPCR analysis of the same sample has repeatedly shown upregulation of this gene). Normally, *AKT* is phosphorylated by *PDPK1,* which is a master kinase activated by several growth factors and hormones that plays an important role in various signaling pathways, including insulin signaling, and is believed to play an important role in regulating the organism size [83]. The increase in *AKT/PKB* expression appears to have inhibited *Glycogen synthase kinase 3 beta (GSK3β)* expression in this study (significantly downregulated, Fig. 5). *GSK3β* was probably inactivated because its expression correlates negatively with *AKT* [84, 85], and *glycogen (starch) synthase (GYS)*. As GYS is an enzyme that catalyzes the final step of glycogen synthesis [86], the inactivation of *GSK3β* by *AKT* promotes glucose storage as glycogen. Thus these results indicate that the HCBD has resulted in increased glycogen synthesis. By promoting glucose storage, insulin inhibited the production and release of glucose in the liver by blocking gluconeogenesis and glycogenolysis [87]. This mechanism is likely to lead to insulin resistance (Fig. 6 and Additional file 1), which is an important component of the pathogenesis of NAFLD in humans, where insulin resistance in adipose tissue leads to increases in circulating glucose and the availability of lipid substrates for the accumulation of hepatic lipids [88].

**Table 2:** Differentially expressed genes in liver transcriptome profiles of control and HCBD groups associated with non-alcoholic fatty liver disease pathway (^a^) and insulin signaling pathway (^b^) in KEGG database

Gene Name	KEGG Number	Nr_annotation	FDR	log2FC	Regulation
SOCS3^a,b^	K04696	Suppressor of cytokine signaling 3b (*Danio rerio*)	0.009144	−2.802	Down
INSR^a,b^	K04527	Insulin receptor a precursor (*Danio rerio*)	0.272547	1.070	Up
IRS^a,b^	K07187	Insulin receptor substrate 2-B-like (*Danio rerio*)	0.013461	1.903	Up
PI3K^a,b^	K02649	Phosphatidylinositol 3-kinase regulatory subunit beta, partial (*Anas platyrhynchos*)	0.048268	−5.230	Down
PDK1^b^	K06276	3-phosphoinositide-dependent protein kinase 1 isoform X1 (*Danio rerio*)	0.050913	1.603	Up
Akt^a,b^	K04456	RAC-gamma serine/threonine-protein kinase (*Danio rerio*)	0.000433	6.580	Up
GSK3^a,b^	K03083	Glycogen synthase kinase-3 beta-like (*Lepisosteus oculatus*)	3.08E-13	−8.657	Down
	K03083	Glycogen synthase kinase-3 beta-like isoform X3 (*Neolamprologus brichardi*)	0.002352	−6.405	Down
AMPK^a,b^	K07198	MAP/microtubule affinity-regulating kinase 3 (*Danio rerio*)	8.08E-13	−8.608	Down
	K07198	MAP/microtubule affinity-regulating kinase 3 (*Esox lucius*)	0.038900	−5.459	Down
	K07198	Serine/threonine-protein kinase MARK1-like (*Stegastes partitus*)	0.012837	−5.540	Down
ACC^b^	K11262	Acetyl-CoA carboxylase 2 isoform X2 (*Danio rerio*)	1	0.369	Up
AP-1^a^	K04448	Transcription factor AP-1 (*Oncorhynchus mykiss*)	5.72E-05	6.614	Up
PEPCK^b^	K01596	Phosphoenolpyruvate carboxykinase (*Acanthopagrus schlegelii*)	0.002169	5.994	Up
FBP^b^	K03841	Fructose-1,6-bisphosphatase 1-like (*Xiphophorus maculatus*)	0.007951	6.039	Up
	K03841	Fructose-1,6-bisphosphatase 1-like isoform X3 (*Cynoglossus semilaevis*)	0.007492	6.142	Up
	K03841	Fructose-1,6-bisphosphatase 1-like (*Poecilia reticulata*)	1.95E-10	−8.129	Down
	K03841	Fructose-1,6-bisphosphatase 1-like (*Oryzias latipes*)	0.020039	−5.485	Down
	K03841	Fructose-1,6-bisphosphatase 1-like (*Poecilia formosa*)	0.040649	−5.577	Down

^a^Non-alcoholic fatty liver disease pathway (ko04932), ^b^Insulin signaling pathway (ko04910)

The expression of *acetyl-CoA carboxylase (ACC)* was significantly upregulated as a result of the downregulation of its inhibitor: *AMP-activated protein kinase (AMPK)* (Figs. 5, 6 and Additional file 1). *AMPK* regulates insulin sensitivity: a continuous reduction in *AMPK* activity is accompanied by insulin resistance, whereas *AMPK* activation increases insulin sensitivity. *AMPK* also controls a number of key players in various metabolic pathways and is therefore a major regulator of glucose and lipid metabolisms [89]. This downregulation of *AMPK* and upregulation of *ACC*, which is crucial for hepatic de novo fatty acid synthesis, are strongly indicative of heightened de novo lipogenesis [61, 90–93]. Thus these results indicate that prolonged exposure of hepatocytes to high glucose concentrations associated with the HCBD has resulted in insulin resistance and increased lipogenesis in liver [94], which is also in agreement with our histological observations. These early symptoms of hepatic metabolic disorders, when prolonged, can lead to a pathological accumulation of TGs in liver and eventually contribute to the onset of fatty liver disease [63, 95, 96].

Annotation results for the *fructose-1,6-bisphosphatase 1 (FBP)* gene indicate the existence of several isoforms, some of which were up- and some down-regulated (Table 2). In mammals, this gene partakes in carbohydrate transport and metabolism via the insulin signaling pathway, which is associated with NAFLD. It plays a role in regulating glucose sensing and insulin secretion of pancreatic beta-cells, and is an important negative regulator of appetite [97]. Some previous studies found that high carbohydrate diets result in increased *FBP* expression [2, 98–100]. However, the existence of several differently regulated paralogs in our results indicates that its regulation mechanism and functions in fish might be more complex than in mammals.

Hepatic *IL6*, which is an important proinflammatory adipocytokine that is almost always significantly elevated in the adipose cells of obese and insulin-resistant individuals and NAFLD patients, was also upregulated in the HCBD group (Fig. 5). Increased lipid accumulation in liver is usually observed in association with elevated expression of this gene [101–103], which is in agreement with the liver histology results in this study. Another gene associated with de novo fatty acid synthesis, *MAX-like protein X (MLX-ChREBP)*, was also upregulated in the HCBD group. Based on many reports that found that *ChREBP-MLX* is a liver transcription factor always responsive to the consumption of high levels of carbohydrates and required for the induction of the expression of lipogenic genes in response to high dietary glucose [104–107], it has been proposed that the activation of *ChREBP-MLX* may be the glucose-dependent mechanism responsible for the synergistic induction of fatty acid synthesis by glucose and insulin [108]. Increased transcription of the *Transcription factor activator protein 1 (AP1)* gene, which was also upregulated in the HCBD group (Fig. 5), has been linked to obesity, hepatic lipid metabolism, and NAFLD [109]. The expression of *interleukin 6 (IL6)*, *transcription factor AP-1 (AP1)*, *phosphoenolpyruvate carboxykinase (PEPCK)* gene was also upregulated in the HCBD group (Table 2, Additional file 1). This is a key enzyme in the synthesis of glucose in liver and kidney, which also plays a role in hepatic energy metabolism [110, 111]. It is not clear why would a gene associated with increased gluconeogenesis be upregulated in association with HCBD. However, glucose level in blood is maintained within well-defined limits, in part due to the precise regulation of *PEPCK* gene expression. For example, overexpression of this enzyme in mice results in symptoms of the type II diabetes mellitus [112]. Because of the importance of the blood glucose homeostasis, a number of hormones regulate a set of genes (including *PEPCK*) in liver, which modulate the rate of glucose synthesis. Thus the upregulation of this gene may be a reflection of hormonal disbalance caused by high glucose levels in blood [113–117].

Correlation between transcriptomic and qPCR results was relatively low: Pearson correlation coefficient (R^2^ = 0.3226), significance analysis (*P* = 0.0428), and linear regression (red line) are shown (Additional file 7). The underlying cause might be different data normalization methods used for qPCR and RNA-seq data analyses. In qPCR, reference genes are used to normalize gene expression, where the expression of target gene is calculated relative to the expression of reference gene. This method assumes that the expression of reference gene(s) is constant for all samples. However in transcriptomic data analysis (RNA-seq), in most cases we assume that each sample has the same total expressed mRNA, which can be expressed as read count per million mapped reads (RPKM). Thus, both individual differences between samples and variation in reference gene expression can cause discrepancies between the results obtained by these two methods. Also, for subtle differences, it is not unlikely that only one method will detect a significant difference in expression, as qPCR and RNA-seq use different scales. Furthermore, in cases where qPCR analysis indicates upregulation, whereas the transcriptomics indicates downregulation, we should not exclude a possibility that the primers designed for qPCR may have non-specifically amplified both (or more) paralogs present in the genome. As a result of a relatively recent (≈8 MYA) genome duplication, teleost fish possess a large number of duplicated genes [118]. Many paralogs have retained their functions [119, 120], and their expression regulation is often very complex [121–123]. Such circumstances may easily result in inconsistent expression analysis results.

## Conclusions

All of our results, which includes HSI values, liver histology, serum biochemistry, serum and liver metabolomics, liver transcriptomics and specific gene expression analyses, show signs of high accumulation of lipids in the liver of blunt snout bream in association with high-carbohydrate diet. Some of the results even indicate pathological liver changes usually associated with NAFLD. Specifically, high levels of LDL, HDL, AST and ALT observed in the serum are all consistent with our hepatic histology results, which indicate excessive lipid accumulation and liver damage. The decline in the liver betaine may be a consequence of pathological hepatic changes associated with the high-carbohydrate diet. Transcriptomic profiles showed that the HCBD had a strong impact on the expression of genes involved in the NAFLD and insulin signaling pathways, which may lead to the development of insulin resistance in hepatocytes and eventually NAFLD. An example is *IL6*, upregulation of which is strongly associated with insulin-resistance and NAFLD. These results are in agreement with previous observations that HCBD causes stress in blunt snout bream [31]. However, although high HSI and hepatic lipid accumulation correlate negatively with hepatic health in fish, further research will be needed to fully assess whether these pathological changes associated with high-carbohydrate diet would actually lead to the development of NAFLD after a longer period of time. Based on our results, we propose that the duration of these future studies should be more than 60 days and that blood serum parameters should be regularly monitored during the feeding trial.

Finally, an additional interesting finding in our study, not directly discussed here, is a very high number of upregulated genes in the HCBD group associated with a number of neurodegenerative diseases (Fig. 4d): Alzheimer’s (145 genes), Huntington’s (131 genes) and Parkinson’s (127 genes). This is a strong indication of neurodegenerative changes associated with high-carbohydrate diet in blunt snout bream, which has been observed in humans as well [124–126]. Therefore, fish might present a good model to study neurodegenerative changes associated with high-carbohydrate diet in humans.

## Additional file

Additional file 1: Genes differentially expressed between HCBD and control groups in two KEGG pathways. (A) Non-alcoholic fatty liver disease pathway (*P* = 0.0564, Enrichment factor = 1.42) and (B) Insulin signaling pathway (*P* = 1, Enrichment factor = 1.13). (DOCX 397 kb)
Additional file 2: Primers designed for qPCR. (DOCX 15 kb)
Additional file 3:
^1^H NMR signal assignment of metabolites in serum and liver extracts. s - singlet; d - doublet; t - triplet; q - quartet; m - multiplet; b - broad peak; dd - doublet of doublets; S - serum; L - liver. (DOCX 13 kb)
Additional file 4: OPLS-DA scores plots and corresponding loading plots. OPLS-DA scores plots (left) and corresponding loading plots (right), derived from ^1^H NMR data, reveal a significant (*r* = 0.602) decrease in the amount of betaine in livers of HCBD group (blue triangles) in comparison to the Control diet group (black squares). The colour code corresponds to the absolute value of the OPLS-DA correlation coefficient |r|, which indicates the contribution of the corresponding variable to the group separation. In these loading plots, the hot-colored (the red end of the spectrum) metabolites contributed more significantly to the intergroup differences than the cold-colored (blue) ones. (DOCX 98 kb)
Additional file 5: The 600 MHz 1H NMR spectra of plasma (P) and liver (L) from control (A) and HCBD (B) groups. The dotted regions were vertically expanded 16 times in the spectra of plasma and liver extracts. The keys for metabolites are given in Additional file 3. (DOCX 3909 kb)
Additional file 6: Top 20 KEGG pathway. (DOCX 14 kb)
Additional file 7: Correlation analysis between transcriptome and qPCR data. Analysis was conducted for the 13 genes associated with NAFLD and insulin signaling pathways (Fig. 5). Transcriptome is log_2_FC (x-axis) and qPCR is 2^-ΔΔCT^ (y-axis). Pearson correlation coefficient (R^2^ = 0.3226), significance analysis (*P* = 0.0428), and linear regression (red line) are shown. (DOCX 29 kb)

## Abbreviations

ACC: Acetyl-CoA carboxylase
AKT: RAC-gamma serine/threonine-protein kinase
ALT: Alanine transaminase
AMPK: AMP-activated protein kinase
AP1: Transcription factor AP-1
AST: Aspartate transaminase
Bp: Basepair
BSB: Blunt snout bream
cDNA: Complementary DNA
ChREBP: MAX-like protein X
DEGs: Differentially expressed genes
FBP: Fructose-1,6-bisphosphatase 1
FDR: False discovery rate
FPKM: Fragments per kilobase million
GO: Gene ontology
GSK3β: Glycogen synthase kinase 3 beta
GYS: Glycogen (starch) synthase
HCBD: High-carbohydrate diet
HDL: High-density lipoprotein
HSI: Hepatosomatic index
IL6: Interleukin 6
INSR: Insulin receptor
IRS: Insulin receptor substrate
KEGG: Kyoto Encyclopedia of Genes and Genomes
KO: KEGG orthology
KOG: Eukaryotic Orthologous Groups
LDL: Low-density lipoprotein
mRNA: Messenger RNA
MYA: Million years ago
NAFLD: Nonalcoholic fatty liver disease
NCBI: National Center for Biotechnology Information
Nr database: Non-redundant database
ORF: Open reading frame
PCR: Polymerase chain reaction
PDK1: Phosphoinositide-dependent protein kinase1
PEPCK: Phosphoenolpyruvate carboxykinase
PI3K: Phosphoinositide-3-kinase
RPKM: Reads per kilobase of exon model per million mapped reads
SNP: Single nucleotide polymorphism
SOCS3: Suppressor of cytokine signaling 3
SSR: Simple sequence repeats or microsatellites
TCA: Tricarboxylic acid
TG: Triglyceride
