# Supplementary material for: Transcriptomics, metabolomics and histology indicate that high-carbohydrate diet negatively affects the liver health of blunt snout bream (Megalobrama amblycephala)
Source: BMC Genomics. 2017 Nov 9;18:856. doi: 10.1186/s12864-017-4246-9 (PMC5680769; doi:10.1186/s12864-017-4246-9)
Supplement: Additional file 1: — Genes differentially expressed between HCBD and control groups in two KEGG pathways. (A) Non-alcoholic fatty liver disease pathway (P = 0.0564, Enrichment factor = 1.42) and (B) Insulin signaling pathway (P = 1, Enrichment factor = 1.13). (DOCX 397 kb) [file 12864_2017_4246_MOESM1_ESM.docx]

**Additional file 1**


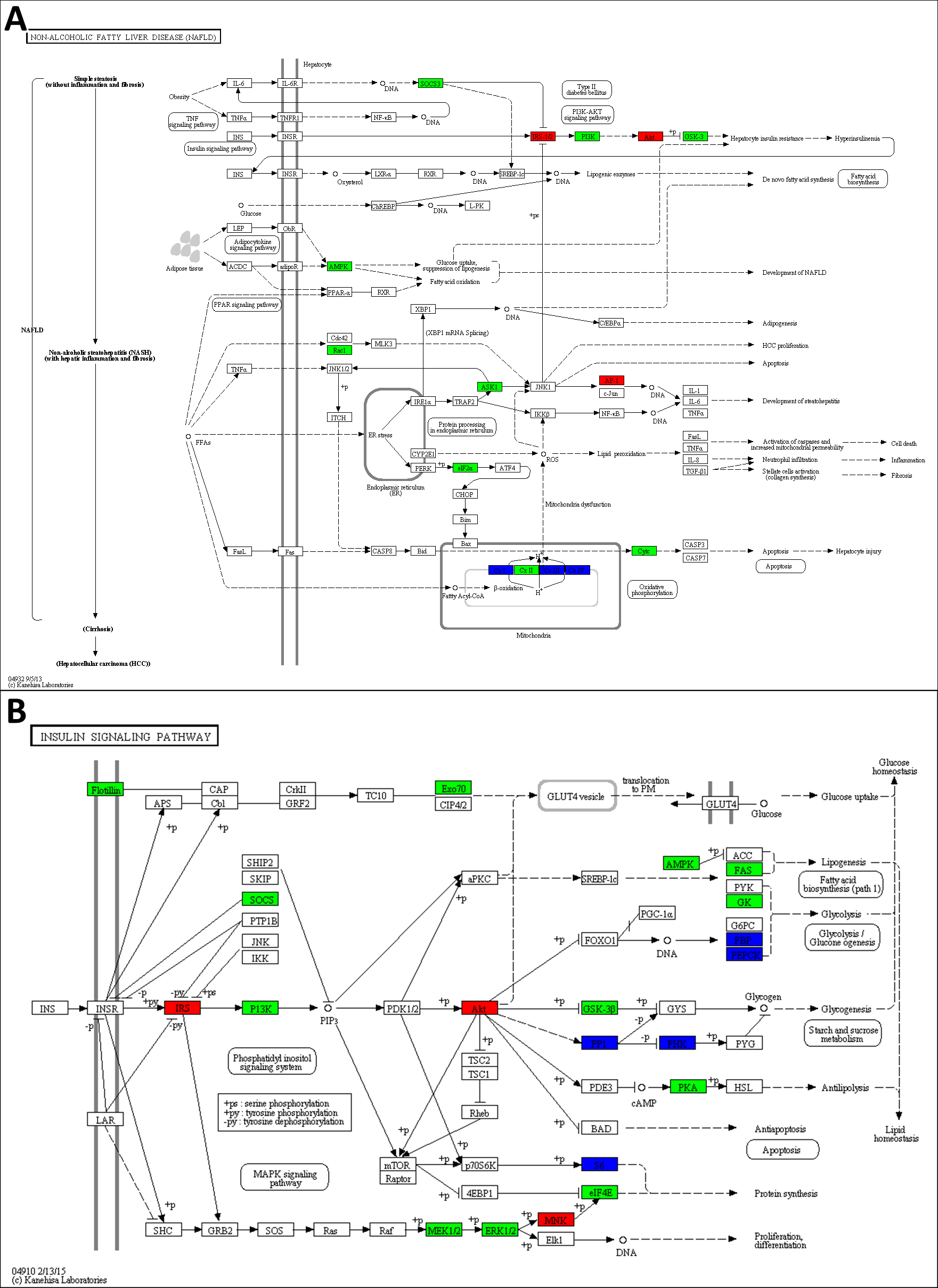


**Figure S3. Genes differentially expressed between HCBD and control groups in two KEGG pathways.**

(A) Non-alcoholic fatty liver disease pathway (*P* =0.0564, Enrichment factor = 1.42) and (B) Insulin signaling pathway (*P* =1, Enrichment factor = 1.13).
