# Supplementary material for: Transcriptomics, metabolomics and histology indicate that high-carbohydrate diet negatively affects the liver health of blunt snout bream (Megalobrama amblycephala)
Source: BMC Genomics. 2017 Nov 9;18:856. doi: 10.1186/s12864-017-4246-9 (PMC5680769; doi:10.1186/s12864-017-4246-9)
Supplement: Additional file 2: — Primers designed for qPCR. (DOCX 15 kb) [file 12864_2017_4246_MOESM2_ESM.docx]

**Additional file 2**

**Table S1. Primers designed for qPCR.**

| **Gene** | **Primer sequence (5’-3’)** | **Tm ºC** |
| --- | --- | --- |
| SOCS3-F | TTCAGTCTGCCTTTTCCTA | 57 |
| SOCS3-R | AAACGGGGTGGCTACTT |  |
| INSR-F | TGAGGACATCACGCACTA | 55 |
| INSR-R | AGCATTGGCTACACCC |  |
| IRS-F | CATTAGCGGCAGGTTGA | 57 |
| IRS-R | CGGTTACGGGACGATTT |  |
| PI3K-F | GTGCGACCTCCTGCTCT | 57 |
| PI3K-R | GCTGCGCTTCCTACTCC |  |
| PDK-F | GTTTAGAGCTGGGAACGA | 60 |
| PDK-R | GCTGGTAGGTAAGGGGTCA |  |
| AKT-F | CCATTCAAACCGCAAGT | 55 |
| AKT-R | CGAACGGCTCCATACC |  |
| GSK3β-F | TTCCTTTGGGATCTGC | 53 |
| GSK3β-R | GGCTCTGTAGTACCGTGA |  |
| GYS-F | CCTCCAGTAACAACTCATAAC | 55 |
| GYS-R | CACAATCTTTACACGGTCA |  |
| AMPK-F | TCATAGACAACCGCCGCATTA | 55 |
| AMPK-R | CCGCCGAACACCGAGAT |  |
| ACC-F | CAGGCTGTCACCTAACTCT | 55 |
| ACC-R | AGCTGCTTCCGTCGTAT |  |
| ChREBP-F | GACATGAGACGCCCTATA | 55 |
| ChREBP-R | AACATCCTCTTCCTGCTT |  |
| IL6-F | GTAGTGGTGGTTTGCCTCA | 53 |
| IL6-R | GCAGCACGGCTTTGTC |  |
| AP1-F | GCGGAGGGCTTTGTGA | 55 |
| AP1-R | GCTGATGGCRGGGTTG |  |
| 18s rRNA-F* | CGGAGGTTCGAAGACGATCA | 60 |
| 18s rRNA-R* | GGGTCGGCATCGTTTACG |  |
| Rpl13a-F | TCTGGAGGACTGTAAGAGGTATGC | 50-57 |
| Rpl13a-R | AGACGCACAATCTTGAGAGCAG |  |

* 18s RNA primer pair was previously published [1].

**References**

1. Tran NT, Gao Z-X, Zhao H-H, Yi S-K, Chen B-X, Zhao Y-H, et al. Transcriptome analysis and microsatellite discovery in the blunt snout bream (Megalobrama amblycephala) after challenge with Aeromonas hydrophila. Fish Shellfish Immunol. 2015;45:72–82.
