# Supplementary material for: Transcriptomics, metabolomics and histology indicate that high-carbohydrate diet negatively affects the liver health of blunt snout bream (Megalobrama amblycephala)
Source: BMC Genomics. 2017 Nov 9;18:856. doi: 10.1186/s12864-017-4246-9 (PMC5680769; doi:10.1186/s12864-017-4246-9)
Supplement: Additional file 3: — 1H NMR signal assignment of metabolites in serum and liver extracts. s - singlet; d - doublet; t - triplet; q - quartet; m - multiplet; b - broad peak; dd - doublet of doublets; S - serum; L - liver. (DOCX 13 kb) [file 12864_2017_4246_MOESM3_ESM.docx]

**Additional file 3**

**Table S2. ^1^H NMR signal assignment of metabolites in serum and liver extracts.**

| **No.** | **Metabolite** | **δ ^1^H (ppm) and multiplicity** | **Proton groups** | **^13^C (ppm)** | **Samples** |
| --- | --- | --- | --- | --- | --- |
| 16 | Succinate | 2.41 (s) | a, β-C**H**_2_ | 37.1 | S, L |
| 27 | Betaine | 3.27 (s)  3.92 (s) | -C**H**_3_  -C**H**_2_ | 56.1  68.7 | S, L |
| 29 | β-Glucose | 3.26 (dd)  3.41(dd)  3.47 (ddd)  3.74 (m)  3.91 (m)  4.65 (d) | 2-C**H**  4-C**H**  5-C**H**  3-C**H**  6-C**H**  1-C**H** | 76.9  72.4  78.7  63.4  63.5  98.8 | S, L |
| 31 | α-Glucose | 3.42 (t)  3.54 (dd)  3.72 (t)  3.77 (m)  3.84 (ddd)  5.24 (d) | 4-C**H**  2-C**H**  3-C**H**  6-C**H**  5-C**H**  1-C**H** | 78.7  74.2  75.4  63.2  74.1  94.9 | S, L |
| 32 | Tyrosine | 3.35 (dd)  6.90 (d)  7.20 (d) | -C**H**  3,5- C**H**  2,6- C**H** | 56.1  118.6  133.4 | S, L |

Notes: s - singlet; d - doublet; t - triplet; q - quartet; m - multiplet; b - broad peak; dd - doublet of doublets; S - serum; L - liver.
