# Supplementary material for: Transcriptomics, metabolomics and histology indicate that high-carbohydrate diet negatively affects the liver health of blunt snout bream (Megalobrama amblycephala)
Source: BMC Genomics. 2017 Nov 9;18:856. doi: 10.1186/s12864-017-4246-9 (PMC5680769; doi:10.1186/s12864-017-4246-9)
Supplement: Additional file 4: — OPLS-DA scores plots and corresponding loading plots. OPLS-DA scores plots (left) and corresponding loading plots (right), derived from 1H NMR data, reveal a significant (r = 0.602) decrease in the amount of betaine in livers of HCBD group (blue triangles) in comparison to the Control diet group (black squares). The colour code corresponds to the absolute value of the OPLS-DA correlation coefficient |r|, which indicates the contribution of the corresponding variable to the group separation. In these loading plots, the hot-colored (the red end of the spectrum) metabolites contributed more significantly to the intergroup differences than the cold-colored (blue) ones. (DOCX 98 kb) [file 12864_2017_4246_MOESM4_ESM.docx]

**Additional file 4**


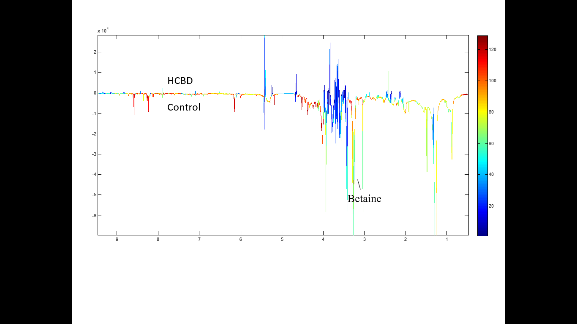


**Figure S1. OPLS-DA scores plots and corresponding loading plots.**

Betaine

OPLS-DA scores plots (left) and corresponding loading plots (right), derived from ^1^H NMR data, reveal a significant (r = 0.602) decrease in the amount of betaine in livers of HCBD group (blue triangles) in comparison to the Control diet group (black squares). The colour code corresponds to the absolute value of the OPLS-DA correlation coefficient |r|, which indicates the contribution of the corresponding variable to the group separation. In these loading plots, the hot-colored (the red end of the spectrum) metabolites contributed more significantly to the intergroup differences than the cold-colored (blue) ones.
