# Supplementary material for: Transcriptomics, metabolomics and histology indicate that high-carbohydrate diet negatively affects the liver health of blunt snout bream (Megalobrama amblycephala)
Source: BMC Genomics. 2017 Nov 9;18:856. doi: 10.1186/s12864-017-4246-9 (PMC5680769; doi:10.1186/s12864-017-4246-9)
Supplement: Additional file 5: — The 600 MHz 1H NMR spectra of plasma (P) and liver (L) from control (A) and HCBD (B) groups. The dotted regions were vertically expanded 16 times in the spectra of plasma and liver extracts. The keys for metabolites are given in Additional file 3. (DOCX 3909 kb) [file 12864_2017_4246_MOESM5_ESM.docx]

**Additional file 5**

P_A_

P_B_

L_A_

L_B_


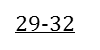


**Figure S2. The 600 MHz ^1^H NMR spectra of plasma (P) and liver (L) from control (A) and HCBD (B) groups.**

The dotted regions were vertically expanded 16 times in the spectra of plasma and liver extracts. The keys for metabolites are given in Additional file 5.
