# Supplementary material for: Transcriptomics, metabolomics and histology indicate that high-carbohydrate diet negatively affects the liver health of blunt snout bream (Megalobrama amblycephala)
Source: BMC Genomics. 2017 Nov 9;18:856. doi: 10.1186/s12864-017-4246-9 (PMC5680769; doi:10.1186/s12864-017-4246-9)
Supplement: Additional file 6: — Top 20 KEGG pathway. (DOCX 14 kb) [file 12864_2017_4246_MOESM6_ESM.docx]

**Additional file 6**

**Table S3. Top 20 KEGG pathway.**

| **Kegg_pathway** | **ko_ID** | **Gene number** | **Enrichment**  **factor** | ***P*-value** |
| --- | --- | --- | --- | --- |
| Ribosome | ko03010 | 269 | 3.46 | 0 |
| Alzheimer's disease | ko05010 | 145 | 1.69 | 1.52E-08 |
| Oxidative phosphorylation | ko00190 | 131 | 1.84 | 0 |
| Huntington's disease | ko05016 | 131 | 1.44 | 0.002195 |
| Parkinson's disease | ko05012 | 127 | 1.77 | 7.53E-09 |
| Carbon metabolism | ko01200 | 119 | 1.57 | 5.61E-05 |
| Tuberculosis | ko05152 | 100 | 1.41 | 0.054536 |
| Non-alcoholic fatty liver disease (NAFLD) | ko04932 | 96 | 1.42 | 0.056437 |
| Neurotrophin signaling pathway | ko04722 | 88 | 1.57 | 0.002218 |
| Protein processing in endoplasmic reticulum | ko04141 | 87 | 1.15 | 1 |
| Phagosome | ko04145 | 83 | 1.32 | 1 |
| Epstein-Barr virus infection | ko05169 | 82 | 1.10 | 1 |
| PI3K-Akt signaling pathway | ko04151 | 81 | 0.78 | 1 |
| Influenza A | ko05164 | 80 | 1.48 | 0.04683 |
| Biosynthesis of amino acids | ko01230 | 74 | 1.46 | 0.117986 |
| Endocytosis | ko04144 | 74 | 0.95 | 1 |
| Glycolysis / Gluconeogenesis | ko00010 | 72 | 1.69 | 0.001104 |
| cGMP-PKG signaling pathway | ko04022 | 68 | 1.00 | 1 |
| Pertussis | ko05133 | 67 | 1.96 | 7.11E-06 |
| Measles | ko05162 | 67 | 1.58 | 0.023656 |
