# Supplementary material for: Transcriptomics, metabolomics and histology indicate that high-carbohydrate diet negatively affects the liver health of blunt snout bream (Megalobrama amblycephala)
Source: BMC Genomics. 2017 Nov 9;18:856. doi: 10.1186/s12864-017-4246-9 (PMC5680769; doi:10.1186/s12864-017-4246-9)
Supplement: Additional file 7: — Correlation analysis between transcriptome and qPCR data. Analysis was conducted for the 13 genes associated with NAFLD and insulin signaling pathways (Fig. 5). Transcriptome is log2FC (x-axis) and qPCR is 2-ΔΔCT (y-axis). Pearson correlation coefficient (R2 = 0.3226), significance analysis (P = 0.0428), and linear regression (red line) are shown. (DOCX 29 kb) [file 12864_2017_4246_MOESM7_ESM.docx]

**Additional file 7**


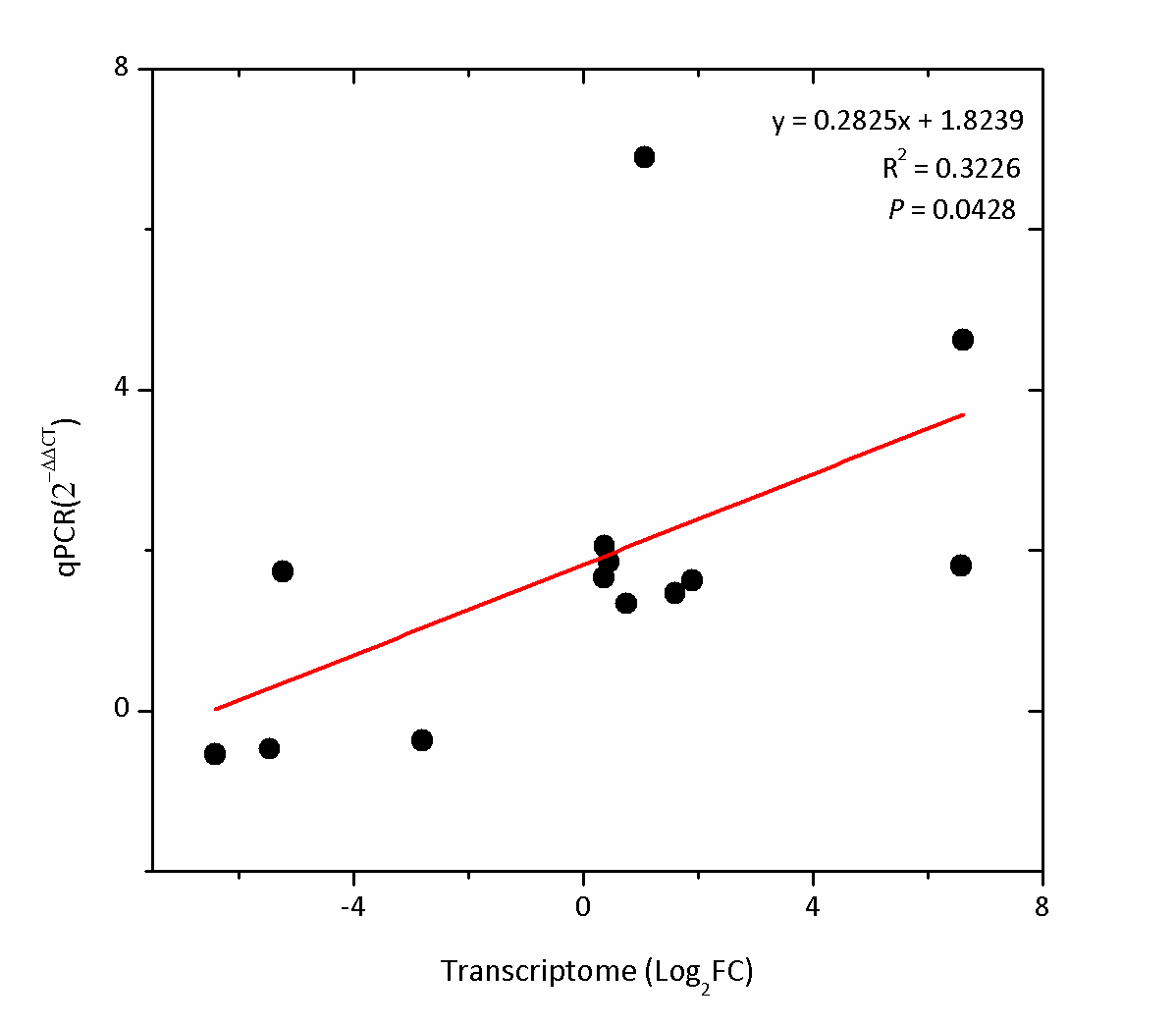


**Figure S4. Correlation analysis between transcriptome and qPCR data.**

Analysis was conducted for the 13 genes associated with NAFLD and insulin signaling pathways (Fig. 5). Transcriptome is log_2_FC (x-axis) and qPCR is 2^-ΔΔCT^ (y-axis). Pearson correlation coefficient (R^2^= 0.3226), significance analysis (*P*= 0.0428), and linear regression (red line) are shown.
